# Incidence Trends in Head and Neck Cancer Subsites: A National Population‐Based Study (2001–2020)

**DOI:** 10.1111/coa.14271

**Published:** 2025-01-12

**Authors:** Kelten Clements, Alex D. McMahon, Lesley Bhatti, Craig Smith, Claire Paterson, Catriona M. Douglas, David I. Conway

**Affiliations:** ^1^ School of Medicine, Dentistry and Nursing University of Glasgow Glasgow UK; ^2^ Public Health Scotland Edinburgh UK; ^3^ Glasgow Head and Neck Cancer (GLAHNC) Research Group Glasgow UK; ^4^ Beatson West of Scotland Cancer Centre Glasgow UK; ^5^ Department of Otolaryngology—Head and Neck Surgery Glasgow Royal Infirmary Glasgow UK; ^6^ Department of Otolaryngology—Head and Neck Surgery Queen Elizabeth University Hospital Glasgow UK; ^7^ Strathclyde Institute of Pharmacy and Biomedical Sciences, University of Strathclyde Glasgow UK

**Keywords:** head and neck cancer, incidence trends, laryngeal cancer, oral cavity cancer, oropharyngeal cancer

## Abstract

**Objectives:**

This descriptive epidemiological study aims to investigate trends in head and neck cancer (HNC) within the anatomical divisions of laryngeal, oropharyngeal, and oral cavity cancers over the past two decades.

**Design:**

Retrospective population‐based observational study.

**Setting:**

Scotland, a constituent country of the United Kingdom, with a population of 5.5 million.

**Participants:**

Newly diagnosed HNC patients in Scotland registered in the Scottish Cancer Registry from 2001 to 2020.

**Main Outcome Measures:**

Trends in age‐standardised incidence rates from 2001 to 2020 for each HNC subsite, anatomical division, and individual sociodemographic using joinpoint regression analysis and Poisson regression analysis.

**Results:**

Overall, HNC incidence rates have remained stable, with an average annual percentage change (AAPC) of 0.29% (*p* = 0.34). However, oropharyngeal cancer showed a significant increase in incidence rates with an AAPC of 3.76% (*p* < 0.001); the tonsils (C09) and the base of the tongue (C01) experienced the greatest increases in AAPC of 4.63% (*p* = 0.001) and 4.79% (*p* < 0.001), respectively. Conversely, laryngeal cancer rates declined significantly, with an AAPC of −2.56% (*p* < 0.001). This decline was primarily influenced by annual reductions of −2.40% (*p* = 0.09) in cancers affecting the glottis (C32.0). Incidence rates for oral cavity cancer remained mostly stable, with an AAPC of −0.60% (*p* = 0.08).

**Conclusion:**

This analysis highlights that behind a stable HNC incidence rate over the past 20 years, there are differential trends among various anatomical divisions with an overall increasing burden of oropharyngeal cancer and declining rates of laryngeal cancer.

**Summary**

- Over the last two decades, the incidence rates for head and neck cancer (HNC) and sociodemographic factors, including age, sex, area‐based socioeconomic deprivation and geographic region, have remained stable.
- A significant increase in oropharyngeal cancer incidence, likely reflects the growing prevalence of HPV‐16‐driven tonsil and base tongue cancers.
- Incidence rates for oral cavity cancer have consistently remained stable throughout the study period.
- Laryngeal cancers have experienced a decline in incidence rates, likely reflecting the declining use of tobacco.
- Awareness of changes in the epidemiology of HNC may allow healthcare providers to plan services appropriately.

## 1 Introduction

Globally, there are more than 660 000 new cases of head and neck cancer (HNC) annually [1], with 12 400 new cases per year in the United Kingdom, making HNC the eighth most common form of cancer in the United Kingdom [2]. However, it is noteworthy that within the United Kingdom, Scotland exhibits significantly higher incidence rates than the national average [2]. The term HNC is an umbrella term and encompasses different subsites, each with distinct presentations, risk factors, and socio‐demographic profiles. Laryngeal cancers are more strongly associated with tobacco use and oral cavity cancers are more strongly associated with alcohol use [3], while oropharyngeal cancers are increasingly caused by human papillomavirus (HPV) infection, particularly HPV‐16 [4]. Correspondingly, different interventions may have varying effects on incidence trends. For example, policies and interventions targeting smoking and alcohol consumption may have impacted specific subsite trends and specific population groups. The landscape of risk factors has further evolved over the past two decades, especially with the emerging oral HPV epidemic [5].

The primary aim of this study was to analyse trends in HNC incidence with a focus on anatomical divisions of the laryngeal, oropharyngeal and oral cavity cancers over the past two decades. For this study, anatomical divisions refer to the specific subgroups within each of the three main subsites. In addition, this study aims to identify the key socio‐demographic determinants behind the observed trends in HNC incidence rates.

## 2 Methods

This retrospective population‐based observational study was conducted according to REporting of studies Conducted using Observational Routinely‐collected Data (RECORD) guidelines. It was approved by the College of Medicine, Veterinary and Life Sciences ethics committee of the University of Glasgow (Project no: 200220043).

### 2.1 Data Collection

Data for all newly diagnosed HNC patients from January 2001 to December 2020 were obtained from the Scottish Cancer Registry, Public Health Scotland [6]. The dataset was of high quality, with 97.4% of HNC cases that were microscopically verified, and only 0.2% relied solely on death certificates for registration [7]. The data included crude counts, population denominators, topographic information on the specific subsite of the tumour, and socio‐demographic information such as age at diagnosis, sex, geographic region and area‐based socio‐economic deprivation measured by the Scottish Index of Multiple Deprivation (SIMD) [8].

### 2.2 Definitions

Age at diagnosis was categorised into three groups: less than 39 years, 40–69 years and 70 years or more.

The SIMD is an area‐based measure of socio‐economic deprivation that is linked to the residential postcodes. These scores are used to categorise the population into five levels, with each category representing 20% of Scotland's population. SIMD 1 corresponds to the most socio‐economically deprived areas, while SIMD 5 represents the least socio‐economically deprived areas. This index is calculated from information gathered from multiple sources relating to seven domains: income; employment; education; health; access to services; crime and housing [8].

Geographical regions are classified into the three distinct regional cancer networks in Scotland. Each network corresponds to the areas served by different NHS health boards: the West of Scotland Cancer Network (WoSCAN) encompassing Ayrshire and Arran, Forth Valley, Greater Glasgow and Clyde and Lanarkshire; the North Cancer Alliance (NCA) covering Grampian, Highland, Orkney, Tayside, Shetland and the Western Isles; and the Southeast Scotland Cancer Network (SCAN) including Borders, Dumfries and Galloway, Fife and Lothian.

Tumour subsites were classified according to the International Classification of Disease for Oncology, 3rd edition (ICD‐O‐3) [9]. It is important to note that different cancer registries and publications have varying definitions for the anatomical divisions related to oropharyngeal and oral cavity cancers. For the current analysis, we adopted previously defined epidemiological groupings [4].

- Oral cavity cancer: Lips (C00), other and unspecified parts of the tongue (C02) (excluding C02.4 lingual tonsils), gums (C03), floor of mouth (C04), palate (C05), other and unspecified parts of the mouth (C06).
- Oropharyngeal cancer: Base of the tongue (C01), lingual tonsil (C02.4), tonsil (C09), oropharynx (C10), other and ill‐defined sites in lips (C14), oral cavity and pharynx.
- Larynx (C32): glottis (C32.0), supraglottis (C32.1), subglottis (C32.2), laryngeal cartilage (C32.3), overlapping lesion of larynx (C32.8), larynx not otherwise specified (NOS) (C32.9).
- Other: Nasopharynx (C11) and hypopharynx (C12 + C13)

### 2.3 Statistical Analysis

To control for variations in age distribution and population size, age‐adjusted incidence rates per 100 000 population were calculated using the new 2013 European standard population [10]. The multivariate Poisson regression analysis was performed using RStudio (Version 2023.03.1, Build 446) to produce rate ratios (RRs), 95% confidence intervals (95% CIs) and the Wald test was used to assess the significance of each coefficient by comparing it to a standard normal distribution. This identified the key socio‐economic and demographic drivers in trends observed in HNC incidence rates. Outputs were considered statistically significant if *p* < 0.05. A joinpoint regression analysis was performed using the Joinpoint Regression Program (Version 5.0.2; National Cancer Institute), a statistical method that identifies points in time where significant changes in rates occur. This was used within each subsite and socio‐demographic factor, assisting in detecting shifts in the linear slope of the trend. Due to the 20‐year time trends investigated, we limited the model to a maximum number of one joinpoint. A Monte Carlo permutation method was used to test the significance of potential models, showing that a model with one joinpoint significantly improved fit, while additional joinpoints did not provide further improvement. The analysis also generated 95% CIs, average annual percentage changes (AAPCs), and annual percentage changes (APCs), which were delineated into two distinct trends denoted as Trend 1 and Trend 2. Trend 1 corresponds to the initial time period spanning from 2001 to the joinpoint, while Trend 2 pertains to the subsequent period from the joinpoint to 2020.

## 3 Results

**FIGURE 1:**
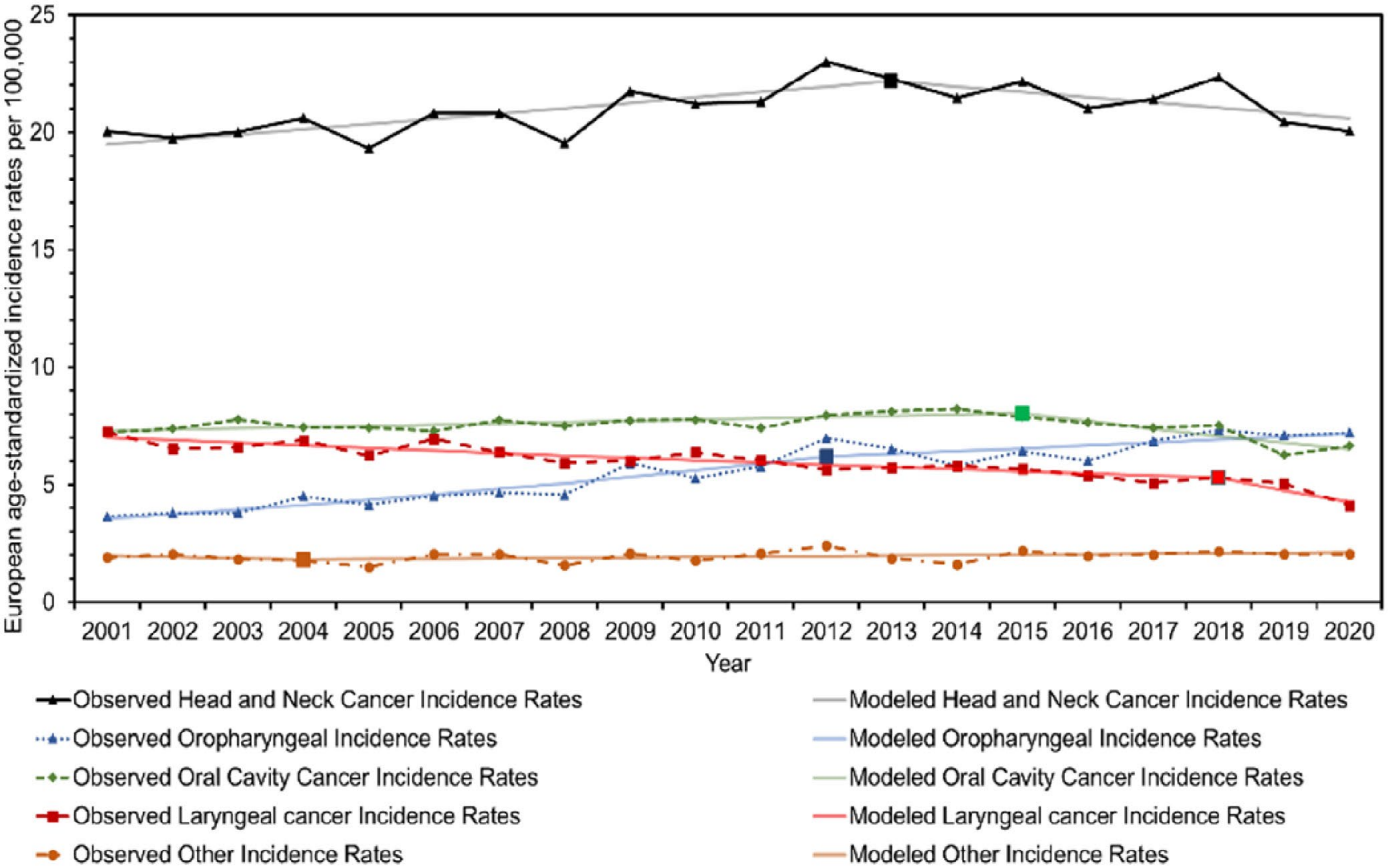
Observed and modelled European age‐standardised incidence rates for the main subsites of head and neck cancer from 2001 to 2020.

**FIGURE 2:**
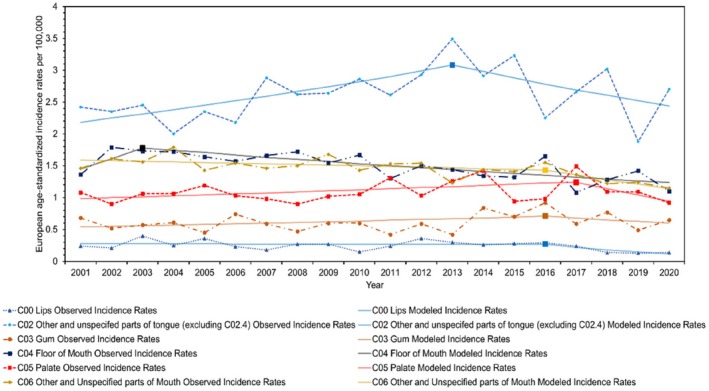
Observed and modelled European age‐standardised incidence rates for each anatomical division of oral cavity cancer from 2001 to 2020.

**FIGURE 3:**
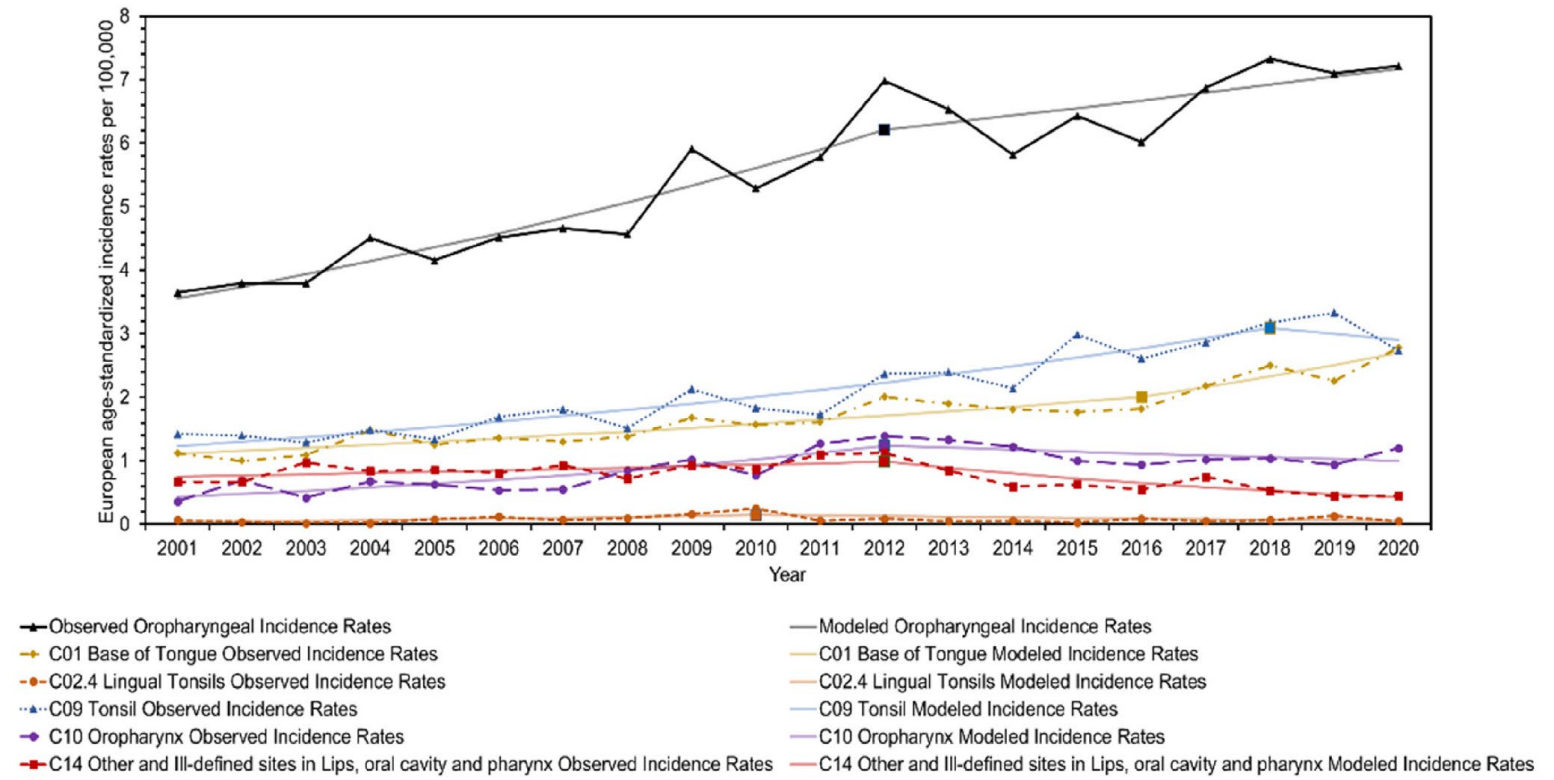
Observed and modelled European age‐standardised incidence rates for each anatomical division of oropharyngeal cancer from 2001 to 2020.

**FIGURE 4:**
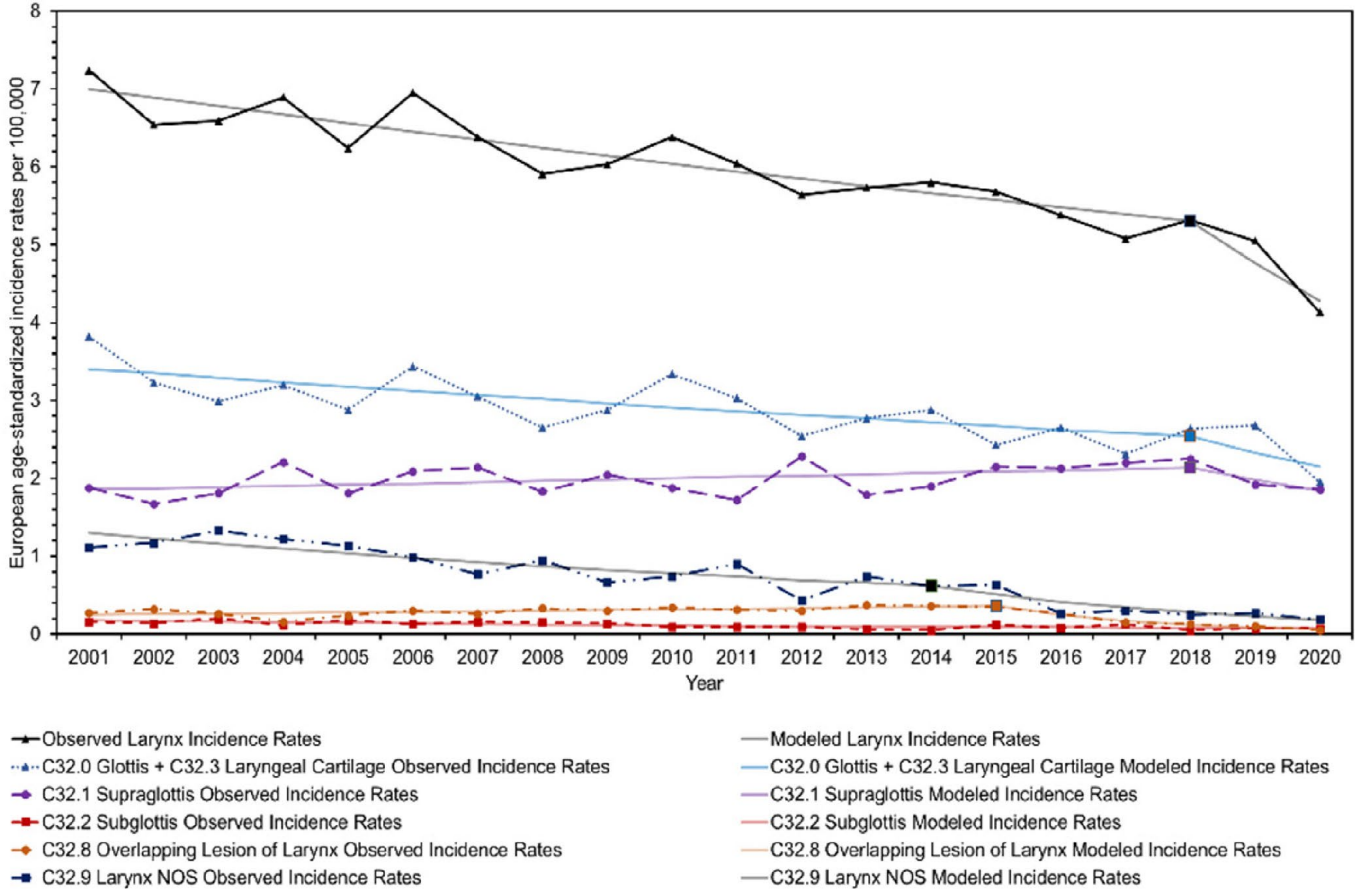
Observed and modelled European age‐standardised incidence rates for each anatomical division of laryngeal cancer from 2001 to 2020.

This study encompassed a total of 20 850 individuals who were diagnosed with HNC in Scotland between 2001 and 2020. Among them, 14 707 (70.5%) were males, 6143 (29.5%) were females and 10 710 (51.4%) were from the WoSCAN region. Table 1 provides an overview of the socio‐demographic characteristics of the study population, as well as the age‐standardised rates and RRs. In addition, graphical representations in Figures 1, 2, 3, 4 and S1–S6, illustrate the changes in age‐standardised incidence rates. These graphs pinpoint the periods identified by the joinpoint regression model as having the most significant changes in rates for each anatomical division of HNC and each socio‐demographic determinant. Detailed descriptions of these findings are provided in the subsequent sections.

**TABLE 1:** Population characteristics for head and neck cancer patients in Scotland between 2001 to 2020.

Characteristic	Count	Population	Age‐standardised incidence rate	Rate ratio (95% CI)	*p*
Overall	20 850	105 251 900	20.96	—	—
SIMD					
SIMD 1	6290	21 080 126	34.46	2.57 (2.45, 2.69)	**< 0.001**
SIMD 2	4867	21 061 673	24.45	1.99 (1.89, 2.09)	**< 0.001**
SIMD 3	3972	21 115 593	19.16	1.62 (1.54, 1.71)	**< 0.001**
SIMD 4	3274	21 126 672	15.89	1.34 (1.27, 1.41)	**< 0.001**
SIMD 5 (ref.)	2447	20 867 836	12.42	—	—
Region					
NCA (ref.)	4821	27 068 610	18.05	—	—
SCAN	5319	29 194 440	19.48	1.10 (1.06, 1.15)	**< 0.001**
WoSCAN	10 710	48 988 850	23.57	2.22 (2.15, 2.30)	**< 0.001**
Age					
≤ 39 (ref.)	414	51 371 957	0.39	—	—
40–69	13 473	41 082 278	12.98	4.36 (3.96, 4.82)	**< 0.001**
≥ 70	6963	12 797 665	7.59	1.41 (1.28, 1.56)	**< 0.001**
Sex					
Male	14 707	50 968 479	32.03	2.92 (2.83, 3.01)	**< 0.001**
Female (ref.)	6143	54 283 421	11.47	—	—
Year					
2001 (ref.)	885	5 064 200	20.03	—	—
2002	881	5 066 000	19.75	1.00 (0.91, 1.10)	0.94
2003	903	5 068 500	20	1.04 (0.95, 1.14)	0.44
2004	933	5 084 300	20.6	1.04 (0.95, 1.14)	0.37
2005	893	5 110 200	19.32	1.00 (0.91, 1.18)	0.99
2006	968	5 133 000	20.81	1.08 (0.99, 1.18)	0.10
2007	987	5 170 000	20.82	1.07 (0.97, 1.17)	0.14
2008	937	5 202 900	19.54	1.01 (0.92, 1.11)	0.82
2009	1058	5 231 900	21.74	1.14 (1.04, 1.24)	**0.005**
2010	1044	5 262 200	21.2	1.08 (0.98, 1.18)	0.09
2011	1062	5 299 900	21.3	1.09 (0.99, 1.19)	0.053
2012	1164	5 313 600	23	1.19 (1.09, 1.30)	**< 0.001**
2013	1139	5 327 700	22.27	1.16 (1.06, 1.27)	**< 0.001**
2014	1111	5 347 600	21.45	1.12 (1.03, 1.23)	**0.01**
2015	1164	5 373 000	22.16	1.17 (1.07, 1.27)	**< 0.001**
2016	1112	5 404 700	21.01	1.10 (1.01, 1.21)	**0.03**
2017	1153	5 424 800	21.4	1.12 (1.03, 1.22)	**0.01**
2018	1217	5 438 100	22.33	1.18 (1.08, 1.28)	**< 0.001**
2019	1126	5 463 300	20.44	1.08 (0.99, 1.18)	0.08
2020	1113	5 466 000	20.05	1.07 (0.98, 1.16)	0.15

### 3.1 Incidence Trends in Socio‐Demographics of HNC Patients Over 20 Years

The Poisson regression analysis revealed significant findings about the incidence rates of HNC. Regarding age, the 40–69 age group had the highest RR of 4.36 (95% CI: 3.96, 4.82), demonstrating that HNC is most prevalent in people between 40 and 69 years. Furthermore, males had a RR more than twice that of females (RR: 2.92, 95% CI: 2.83, 3.01), indicating a higher incidence among males. In addition, the analysis revealed a relationship between socio‐economic deprivation and HNC incidence rates, with higher rates in more socio‐economic deprived areas (Table 1).

To analyse the dynamic and quantitative trends in HNC incidence rates over the past two decades, a joinpoint regression analysis was conducted. This revealed no statistically significant changes in any of the socio‐demographic determinants investigated during this period (Table 2; Figures S2–S6).

**TABLE 2:** Joinpoint regression analysis of trends in age‐standardised incidence rates of socio‐demographics characteristics, main subsites and anatomical divisions of head and neck cancer patients between 2001 to 2020.

Characteristic	Trend 1^a^	Annual percent change (APC) (95% CI)	*p*	Trend 2^b^	Annual percent change (APC) (95% CI)	*p*	Average annual percent change (AAPC) (95% CI)	*p*
Overall	2001–2013	1.09 (0.44, 1.74)	**0.002**	2013–2020	−1.05 (−2.40, 0.31)	0.12	0.29 (−0.30, 0.89)	0.34
Sex								
Male	2001–2018	0.45 (0.08, 0.82)	**0.02**	2018–2020	−6.31 (−15.11, 3.40)	0.18	−0.29 (−1.28, 0.72)	0.57
Female	2001–2012	1.46 (0.52, 2.41)	**0.004**	2012–2020	−0.95 (−2.31, 0.44)	0.17	0.44 (−0.29, 1.18)	0.24
Age group								
≤ 39 years	—	—	—	—	—	—	1.17 (−3.25, 5.79)	0.61
40–69 years	—	—	—	—	—	—	0.16 (−0.64, 0.96)	0.70
≥ 70 years	2001–2015	1.47 (0.94, 2.01)	**< 0.001**	2015–2020	−2.54 (−4.77, −0.27)	**0.03**	0.40 (−0.26, 1.07)	0.24
SIMD								
1—Most deprived	2001–2018	0.83 (0.19, 1.48)	**0.01**	2018–2020	−8.47 (−23.60, 9.65)	0.31	−0.19 (−2.00, 1.65)	0.83
2	—	—	—	—	—	—	0.12 (−1.30, 1.55)	0.87
3	—	—	—	—	—	—	0.42 (−1.38, 2.25)	0.65
4	2001–2015	1.62 (0.61, 2.63)	**0.003**	2015–2020	−2.63 (−6.89, 1.82)	0.22	0.48 (−0.79, 1.77)	0.46
5—Least deprived	—	—	—	—	—	—	0.58 (−0.82, 1.99)	0.42
Geographical location								
NCA	2001–2015	1.33 (0.21, 2.46)	**0.02**	2015–2020	−3.27 (−7.99, 1.71)	0.18	0.09 (−1.32, 1.54)	0.89
SCAN	—	—	—	—	—	—	0.36 (−1.08, 1.83)	0.62
WoSCAN	2001–2015	0.80 (0.17, 1.45)	**0.02**	2015–2020	−1.25 (−4.03, 1.60)	0.36	0.26 (−0.55, 1.08)	0.53
**Oral cavity cancer**	2001–2015	0.69 (0.17, 1.21)	**0.01**	2015–2020	−4.11 (−6.41, −1.77)	**0.002**	−0.60 (−1.28, 0.08)	0.08
Lip (C00)	—	—	—	—	—	—	−4.23 (−9.63, 1.50)	0.14
Other and unspecified parts of the tongue (C02) (excluding C02.4)	2001–2013	2.90 (0.55, 5.31)	**0.02**	2013–2020	−3.27 (−7.77, 1.45)	0.16	0.58 (−1.51, 2.72)	0.59
Gum (C03)	—	—	—	—	—	—	0.58 (−3.46, 4.79)	0.78
Floor of mouth (C04)	2001–2003	10.60 (−18.71, 50.49)	0.50	2003–2020	−2.11 (−3.14, −1.07)	**0.001**	−0.85 (−3.88, 2.28)	0.59
Palate (C05)	—	—	—	—	—	—	−0.22 (−3.27, 2.92)	0.89
Other and unspecified parts of mouth (C06)	—	—	—	—	—	—	−1.73 (−3.26, −0.17)	**0.03**
**Oropharyngeal cancer**	2001–2012	5.18 (3.70, 6.68)	**< 0.001**	2012–2020	1.83 (0.04, 3.65)	**0.04**	3.76 (2.70, 4.82)	**< 0.001**
Base of tongue (C01)	2001–2016	4.02 (2.70, 5.36)	**< 0.001**	2016–2020	7.72 (0.56, 15.38)	**0.04**	4.79 (3.10, 6.51)	**< 0.001**
Lingual tonsil (C02.4)	2001–2010	14.50 (2.77, 27.57)	**0.02**	2010–2020	−8.77 (−16.51, 0.32)	**0.04**	1.59 (−4.68, 8.27)	0.63
Tonsil (C09)	2001–2018	5.54 (4.32, 6.78)	**< 0.001**	2018–2020	−2.87 (−24.50, 24.96)	0.81	4.63 (1.92, 7.40)	**0.001**
Oropharynx (C10)	2001–2012	10.04 (5.55, 14.71)	**< 0.001**	2012–2020	−2.65 (−7.31, 2.24)	0.26	4.50 (1.50, 7.60)	**0.003**
Other and ill‐defined sites in lip, oral cavity, and pharynx (C14)	2001–2012	2.54 (−0.24, 5.39)	0.07	2012–2020	−9.97 (−14.34, −5.38)	**< 0.001**	−2.93 (−5.25, −0.55)	**0.016**
**Larynx**	2001–2018	−1.63 (−2.06, −1.19)	**< 0.001**	2018–2020	−10.66 (−21.66, 3.03)	0.12	−2.56 (−3.89, −1.21)	**< 0.001**
Glottis and laryngeal cartilage (C32.0 + C32.3)	2001–2018	−1.72 (−2.62, −0.81)	**0.001**	2018–2020	−7.99 (−30.52, 21.54)	0.54	−2.40 (−5.12, 0.40)	0.09
Supraglottis (C32.1)	—	—	—	—	—	—	−0.06 (−2.70, 2.66)	0.97
Subglottis (C32.2)	2001–2013	−5.77 (−10.01, −1.32)	**0.01**	2013–2020	−1.53 (−13.12, 11.61)	0.80	−4.23 (−8.92, 0.70)	0.09
Overlapping lesions of larynx (C32.8)	2001–2015	2.55 (0.54, 4.59)	**0.02**	2015–2020	−30.47 (−39.62, −19.92)	**< 0.001**	−7.42 (−10.75, −3.96)	**< 0.001**
Larynx not otherwise specified (C32.9)	2001–2014	−5.54 (−8.09, −2.93)	**< 0.001**	2014–2020	−18.33 (−28.60, −6.59)	**0.01**	−9.78 (−13.55, −5.85)	**< 0.001**
**Other**	—	—	—	—	—	—	0.35 (−2.57, 3.36)	0.82
Nasopharynx (C11)	—	—	—	—	—	—	2.69 (−1.20, 6.72)	0.18
Hypopharynx (C12 + C13)	—	—	—	—	—	—	−0.06 (−2.89, 2.85)	0.97

*Note*: Excludes statistically insignificant average annual percentage changes.

^a^ Represents the estimated annual percent change (APC) in head and neck cancer incidence for the initial specified time period, ranging from 2001 to the joinpoint.

^b^ Represents the estimated annual percent change (APC) in head and neck cancer incidence for the subsequent specified time period, ranging from the joinpoint to 2020.

### 3.2 Incidence Trends in Anatomical Divisions of HNC Over 20 Years

Over the past 20 years, the overall incidence rates of HNC have remained relatively stable, with a small AAPC of 0.29% (95% CI: −0.30, 0.89) (*p* = 0.34) (Table 2; Figure 1). However, notable changes have occurred in the incidence trends of HNC subsites during this period. Oropharyngeal cancer has shown a significant increase in incidence rates, with an average yearly growth of 3.76% (95% CI: 2.70, 4.82) (*p* < 0.001) (Table 2; Figure 3). Since 2012, it has surpassed the number of laryngeal cancer cases, and since 2019, it has also exceeded oral cavity cancer cases, making it the subsite with the highest incidence rates among the three. Conversely, laryngeal cancer has experienced a significant decline in incidence rates, with an average yearly decline of −2.56% (95% CI: −3.89, −1.21) (*p* < 0.001) (Table 2; Figure 4). Meanwhile, incidence rates for oral cavity cancer have mostly remained stable, with an AAPC of −0.60% (95% CI: −1.28, 0.08) (*p* = 0.08) (Table 2; Figure 2).

While various anatomical divisions of oral cavity cancer exhibited minor fluctuations, only other and unspecified parts of the mouth (C06) demonstrated a significant average yearly change, declining by −1.73% (*p* = 0.03) per year. Overall incidence rates for oral cavity cancer did not experience significant changes (Table 2; Figure 2).

Tonsils (C09) were the most common site for oropharyngeal cancer from 2001 to 2019, with an average increase of 4.63% (*p* = 0.001) per year. The base of the tongue (C01) was the second most common site, which followed a similar upward trend with an average increase of 4.79% (*p* < 0.001) per year. However, in 2020, the base of the tongue (C01) overtook the tonsils (C09), becoming the most common site for oropharyngeal cancer. Despite being anatomically related to the base of the tongue (C01), the lingual tonsils (C02.4) did not exhibit a significant shift in incidence rates. The oropharynx (C10) moved up to the third most common site, with an average increase of 4.50% (*p* = 0.003) per year. While C14, which includes unspecified tumour locations in the pharynx and Waldeyer's ring, decreased in incidence rates with an average decline of −2.93% (*p* = 0.016) per year (Table 2; Figure 3).

Among the laryngeal subsites, glottis (C32.0) was the most common location, but its incidence rate declined by an average of −2.40% (*p* = 0.09) per year. Supraglottis (C32.1) was the second most common location and had a stable incidence rate over 20 years, with the smallest average decline of −0.06% (*p* = 0.97) per year. Larynx NOS (C32.9) showed a significant decline in incidence rates, with an average decline of −9.78% (*p* < 0.001) per year. The lowest incidence rates were observed in overlapping lesions of the larynx (C32.8) and subglottis (C32.2), with 0.05 and 0.07 cases per 100 000 in 2020 (Table 2; Figure 4).

## 4 Discussion

HNC is a complex disease comprising distinct subsites, each exhibiting unique presentations and associated risk factors. This study found that overall incidence rates have remained stable in Scotland over the past two decades, with only minor changes in socio‐demographic factors. This contrasts with the previously reported trend of consistent increases in HNC rates in Scotland during the 1990s and early 2000s [11]. However, there have been notable shifts in the incidence rates of specific subsites and anatomical divisions of HNC, which may indicate changes in risk factors and the impact of socio‐economic and demographic factors.

Oropharyngeal cancer has shown an alarming rise in incidence rates, largely attributed to an increase in tonsil and base tongue cancers. This increase is likely due to HPV‐16‐associated cancers, as the virus preferentially infects these areas [4, 12]. The implementation of HPV vaccination programs has shown promise in reducing the burden of other HPV‐related cancers [13]. However, the impact on oropharyngeal cancer is not yet evident in this study's timeframe and will likely become clearer over the next 30–40 years [14].

Laryngeal cancer, often linked to tobacco use [3], has seen a decline in incidence rates each year over the past two decades. Our analysis showed that this decline is primarily driven by a reduction in glottis cases, whereas supraglottis incidence rates have remained relatively stable. Glottis and supraglottis cancers have distinct profiles, with supraglottic cancer more likely to present in women and at an advanced stage [15]. Several case–control studies have indicated that the risk associated with tobacco use is higher for supraglottic cancer compared to glottic cancers [16, 17]. This suggests that tobacco control programs, which have led to a 17% reduction in cigarette consumption over the past two decades in Scotland [18], may not fully explain the observed trends in anatomical divisions of laryngeal cancer. In addition, improvements in cancer registration practices may have reduced cases categorised as larynx NOS (C32.9), redistributing these cases to specific anatomical divisions and potentially influencing observed trends. Interestingly, studies have found that supraglottic cancer has a higher risk of HPV positivity than glottic cancer [19]. This suggests that while the decline in tobacco use as a risk factor may have contributed to the reduction in glottis cancer cases, the stable incidence rates of supraglottis cancer could be attributed to a corresponding increased risk of HPV infections. Another potential contributing factor to this divergent trend could be a high prevalence of recreational drug misuse, with a local retrospective HNC cohort study reporting an increased risk of supraglottic cancer among those using recreational drugs compared to those who did not use drugs [20].

Despite an 11% decrease in harmful alcohol consumption in Scotland over the past two decades, with the mean number of alcohol units consumed dropping from 16.2 in 2003 to 11.3 in 2021 [18], oral cavity cancer, which is strongly linked to alcohol consumption [3], has exhibited stable incidence rates. A large international pooled analysis study showed that the beneficial effect of quitting alcohol on cancer risk is not observed for more than 20 years after quitting, unlike quitting smoking, which showed beneficial effects within 1–4 years [21]. This difference may explain why the decline in smoking rates has been reflected by a decline in laryngeal cancer rates, but the decline in alcohol consumption has not yet resulted in a corresponding reduction in oral cavity cancer rates.

Socio‐economic factors have a substantial influence on HNC incidence rates, which show a consistent correlation with socio‐economic inequalities. People from socio‐economically deprived areas face challenges in accessing healthcare services and have higher rates of smoking and alcohol consumption in their communities [22, 23, 24]. The spatial disparity observed in incidence rates mirrors areas of deprivation, with 9 out of the top 10 most impoverished regions in Scotland being within the region with the highest incidence rates—the WoSCAN region [25]. However, relying solely on area‐based socio‐economic indicators, such as SIMD, has limitations and may underestimate the health associations [26]. Future research should incorporate individual‐level socio‐economic measures to better understand HNC risk factors and develop targeted interventions.

Our study utilised a population‐level dataset to analyse trends over two decades. However, certain limitations should be considered. We were unable to evaluate individual‐level risk factors such as HPV status, smoking history, and alcohol consumption. In addition, although the number of paediatric HNC cases included in this study was small, these cases constitute a distinct entity characterised by unique risk profiles compared to adult HNC [27]. Finally, the impact of emerging risk factors, including vaping and exposure to novel carcinogens, on HNC incidence is not fully understood and warrants further investigation.

## 5 Conclusion

Our analysis has revealed distinct trends in the HNC incidence across specific anatomical divisions. The stable socio‐economic profile, strongly associated with deprivation, highlights the importance of addressing socio‐economic disparities in combating HNC. While public health measures such as those to reduce smoking may already be impacting laryngeal cancer incidence, the effects of others, such as the HPV vaccination programs and reduction in alcohol consumption, are yet to affect oropharyngeal and oral cancer rates, respectively. To fully understand the underlying dynamics, further research is warranted, including individual‐level risk factors. This will pave the way for a more refined and targeted approach to HNC prevention, diagnosis, and treatment.

## Author Contributions

**Kelten Clements:** writing the first draft manuscript and statistical analysis. **David I. Conway:** conception and design. **Alex D. McMahon:** statistical advice. **Craig Smith:** data specification. **Lesley Bhatti:** data provision. All authors: critical revision of the article.

## Ethics Statement

This study was approved by the MVLS college ethics committee of the University of Glasgow (Project no: 200220043).

## Consent

The authors have nothing to report.

## Conflicts of Interest

The authors declare no conflicts of interest.

### Peer Review

The peer review history for this article is available at https://www.webofscience.com/api/gateway/wos/peer‐review/10.1111/coa.14271.

## Supporting information


**Data S1.** Supporting Information.

## Data Availability

Data sharing is not applicable because this study involved the analysis of an existing database and did not generate any new data.
