# Supplementary material for: Incidence Trends in Head and Neck Cancer Subsites: A National Population‐Based Study (2001–2020)
Source: Clin Otolaryngol. 2025 Jan 12;50(3):474–84. doi: 10.1111/coa.14271 (PMC11975201; doi:10.1111/coa.14271)
Supplement: Supplementary file 1 — Data S1. Supporting Information. [file COA-50-474-s001.docx]

**Supplementary Material**

***Figure S1*** *Observed and modelled European age-standardised incidence rates of the other head and neck cancer subsites from 2001 to 2020.*


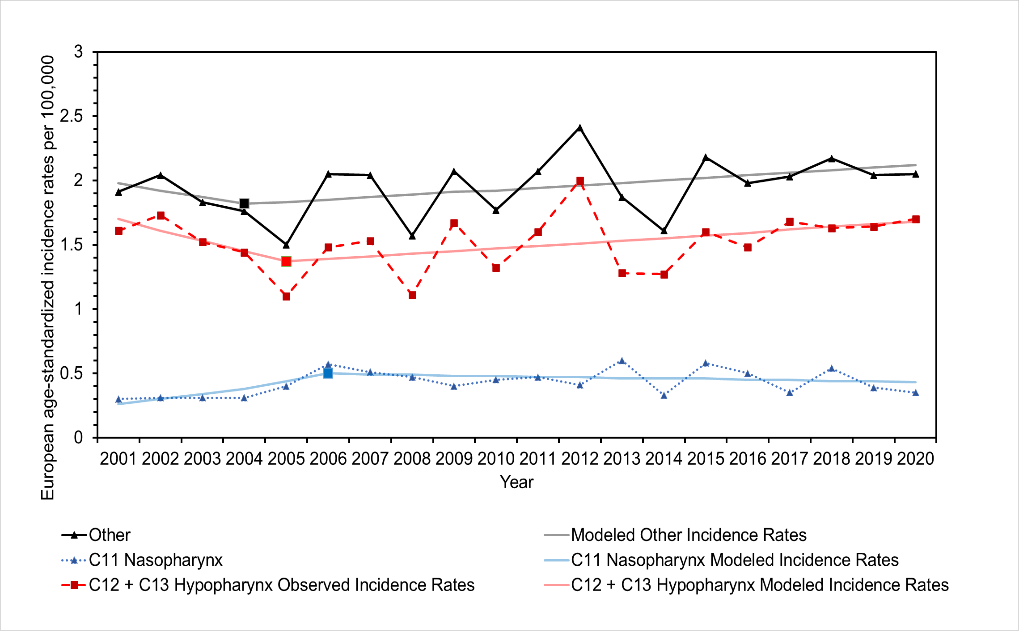


***Figure S2*** *Observed and modelled European age-standardised incidence rates for each geographic region of Scotland for head and neck cancer from 2001 to 2020.*


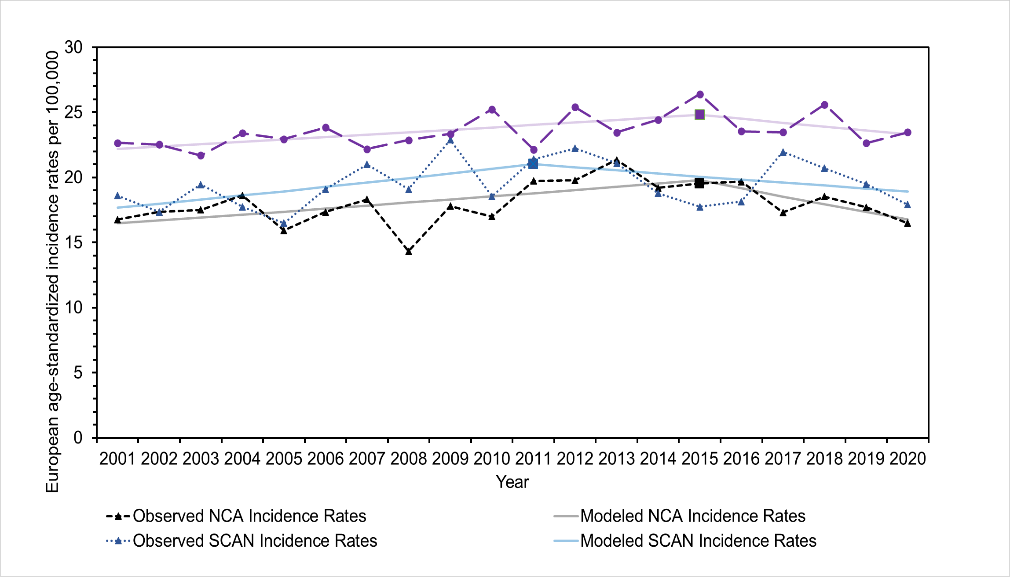


***Figure S3*** *Observed and modelled European age-standardised incidence rates for each SIMD quintile level for head and neck cancer from 2001 to 2020.*


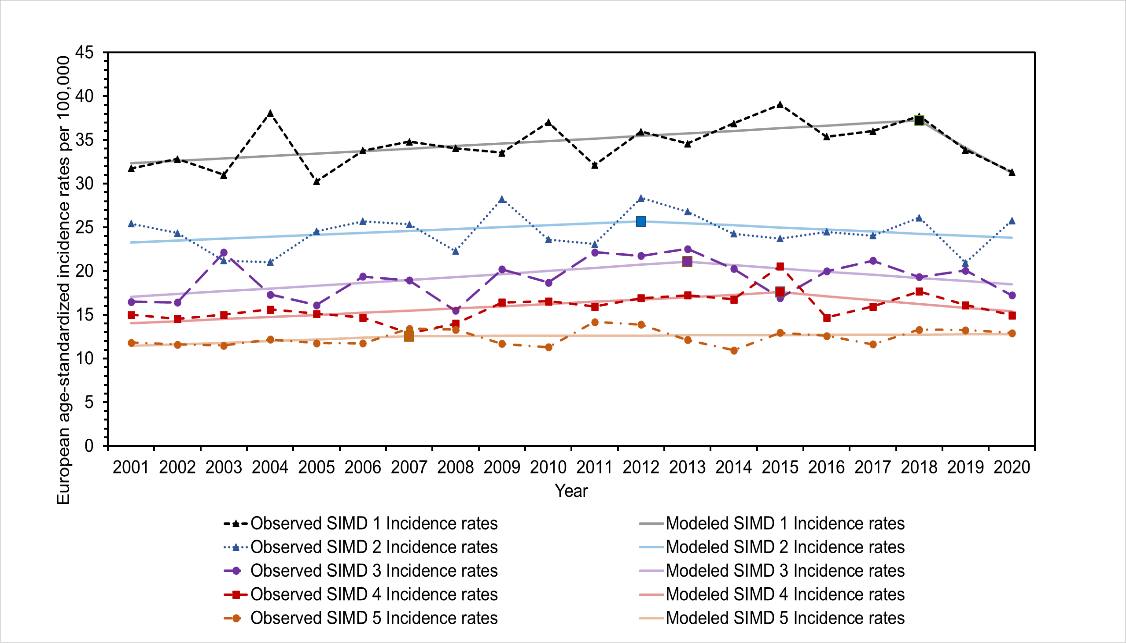


***Figure S4*** *Geographic variations in the European age-standardised incidence rate in Scotland from 2001 to 2020.*


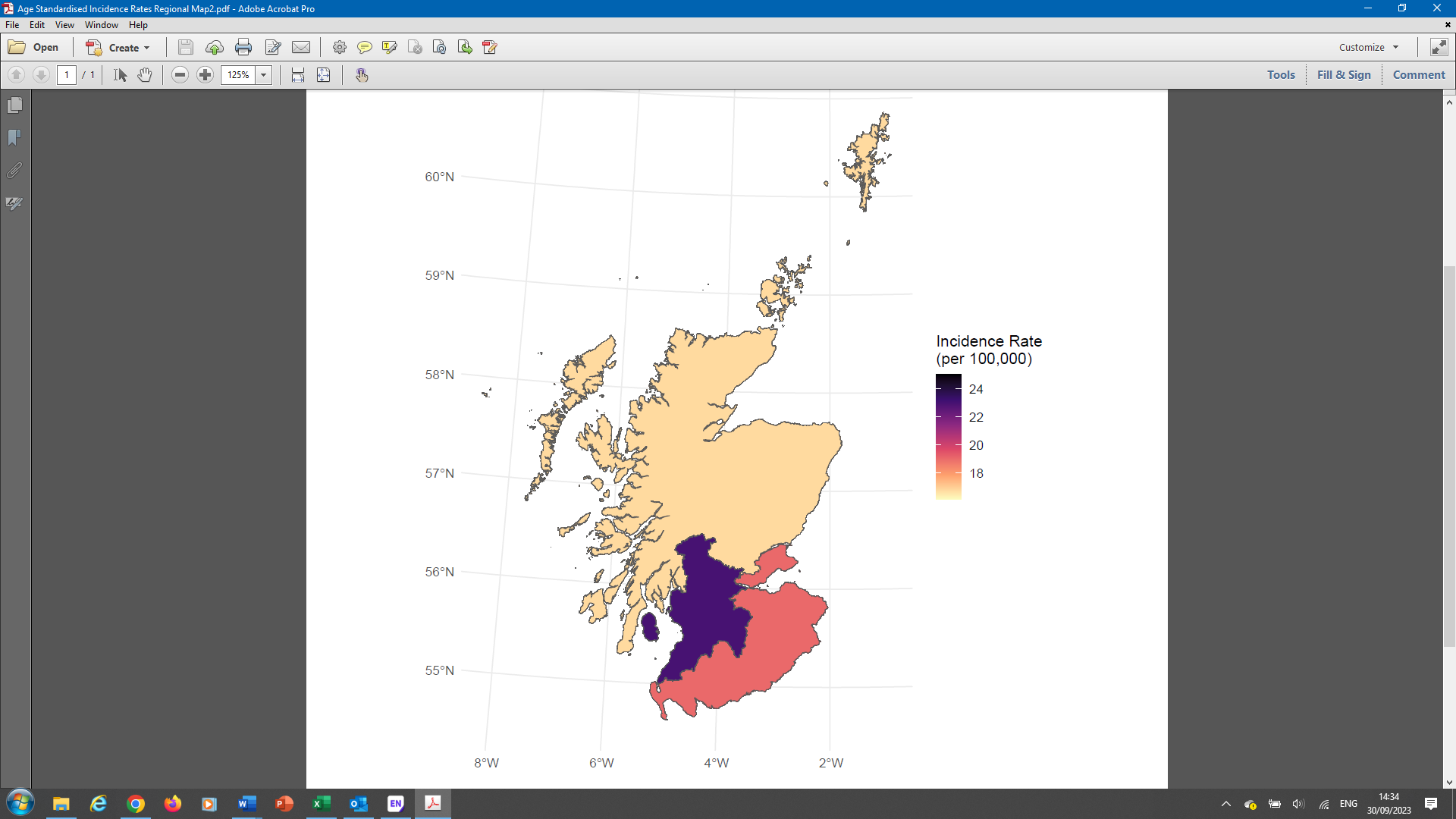


***Figure S5*** *Observed and modelled European age-standardised incidence rates for each age group for head and neck cancer from 2001 to 2020.*

*
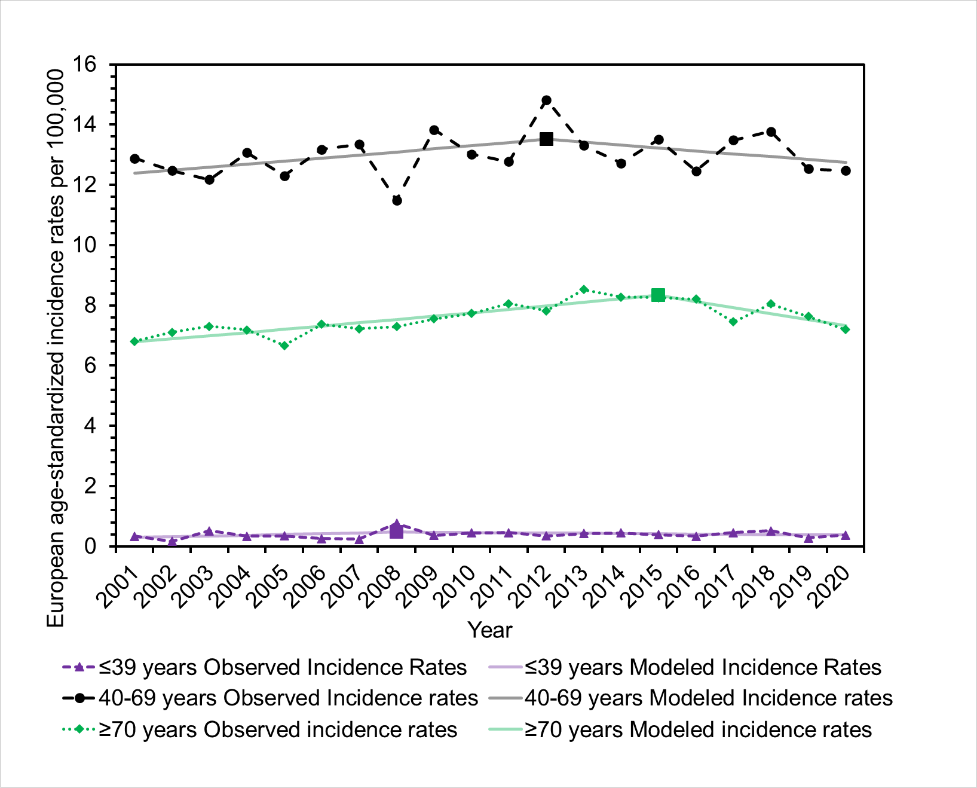
*

***Figure S6*** *Observed and modelled European age-standardised incidence rates for both sexes for head and neck cancer from 2001 to 2020.*

**
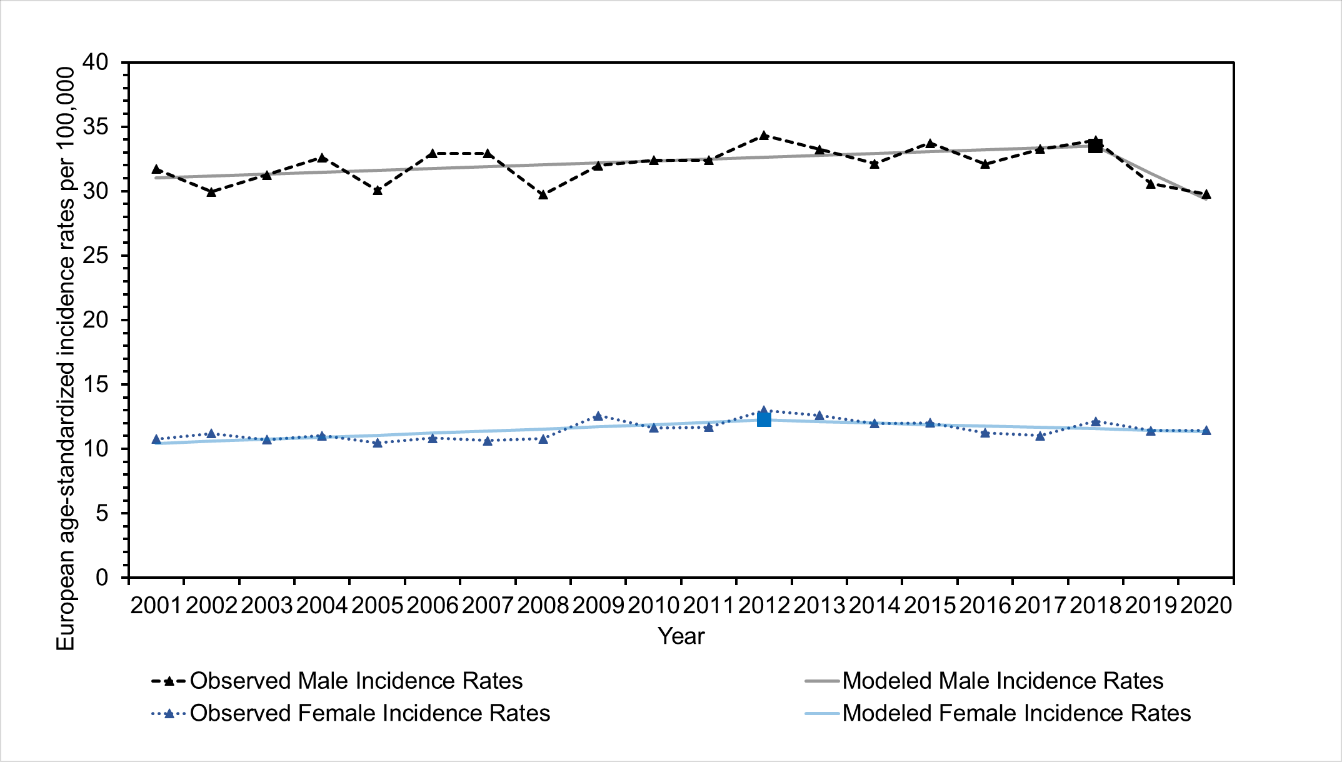
**
